# Draft genome sequence of a member of a putatively novel *Rubrobacteraceae* genus from lava tubes in Lava Beds National Monument

**DOI:** 10.1128/mra.01335-24

**Published:** 2025-04-21

**Authors:** Catherine Maggiori, Zachariah John, Dina M. Bower, Maëva Millan, Aria S. Hahn, Amy McAdam, Sarah Stewart Johnson

**Affiliations:** 1 Department of Biology, Georgetown University 8368, Washington, DC, USA; 2 Department of Astronomy, University of Maryland 248531 , College Park, Maryland, USA; 3 Planetary Systems Laboratory, NASA Goddard Space Flight Center 53523, Greenbelt, Maryland, USA; 4 LATMOS Laboratoire Atmosphères, Chargée de recherche (CNRS) 27051 , Guyancourt, Île-de-France, France; 5 Koonkie Inc. 53523, Vancouver, British Columbia, Canada; 6 Planetary Environments Laboratory, NASA Goddard Space Flight Center 53523, Greenbelt, Maryland, USA; DOE Joint Genome Institute, Berkeley, California, USA

**Keywords:** microbiology, microbial ecology, astrobiology, metagenomics, environmental microbiology

## Abstract

We report the draft genome sequence of a member of a potentially novel genus of *Rubrobacteraceae* isolated from Golden Dome Cave in Lava Beds National Monument. Members of this family are known to inhabit thermophilic environments. The metagenome-assembled genome presented here helps illuminate the genetic capacity of basaltic lava tube environments.

## ANNOUNCEMENT

Lava Beds National Monument contains numerous volcanic formations, such as basaltic lava tubes analogous to those on Mars. Golden Dome is one such cave, composed of lava flow from the Medicine Lake volcano ~36,000 years ago (1). Rock samples from the walls of Golden Dome cave were collected aseptically on 10 May 2022, frozen, and shipped to Georgetown University. The samples were stored at −80°C until analysis.

Samples underwent a pre-extraction chemical lysis: ~250 mg of sample was mixed with 400 µL 2× PBS, 25 µL metapolyzyme (cat. no. MAC4LDF-5 X1VL), and 250 µL ZymoBIOMICS DNA Miniprep Kit Lysis Solution and vortexed for 10 seconds. The mixture was incubated at 35°C for 2 hours, with intervals of 10 second vortexing every 30 minutes. 15 µL of proteinase K was then added, followed by another incubation at 55°C for 1 hour. DNA was extracted from the lysed samples using steps 4–11 of the ZymoBIOMICS DNA Miniprep Kit (cat. no. D6035).

A paired-end library was constructed using 3.12 ng of DNA and Illumina’s Nextera XT DNA Library Preparation Kit with Illumina DNA/RNA UD Indexes and sequenced on a NovaSeq platform to produce 5,017,368 150 bp reads. Reads were trimmed to remove adapters and low-quality bases using Trimmomatic version 0.39 (2). The remaining 5,015,316 reads were assembled using SPAdes genome assembler version 4.0.0 (metaSPAdes mode) (3) and filtered for a minimum length of 500 bp using bbtools version 39.08. Bins were generated using MetaBat2 version 2.17 (4), quality checked with CheckM version 1.2.3 (5), and analyzed with anvi’o version 8 (6). KEGG KOfam and COG databases were used for annotation, and GTDb-tk version 2.4.0 was used to assign taxonomy (7). All tools were used with default parameters, unless otherwise specified.

**Fig 1:**
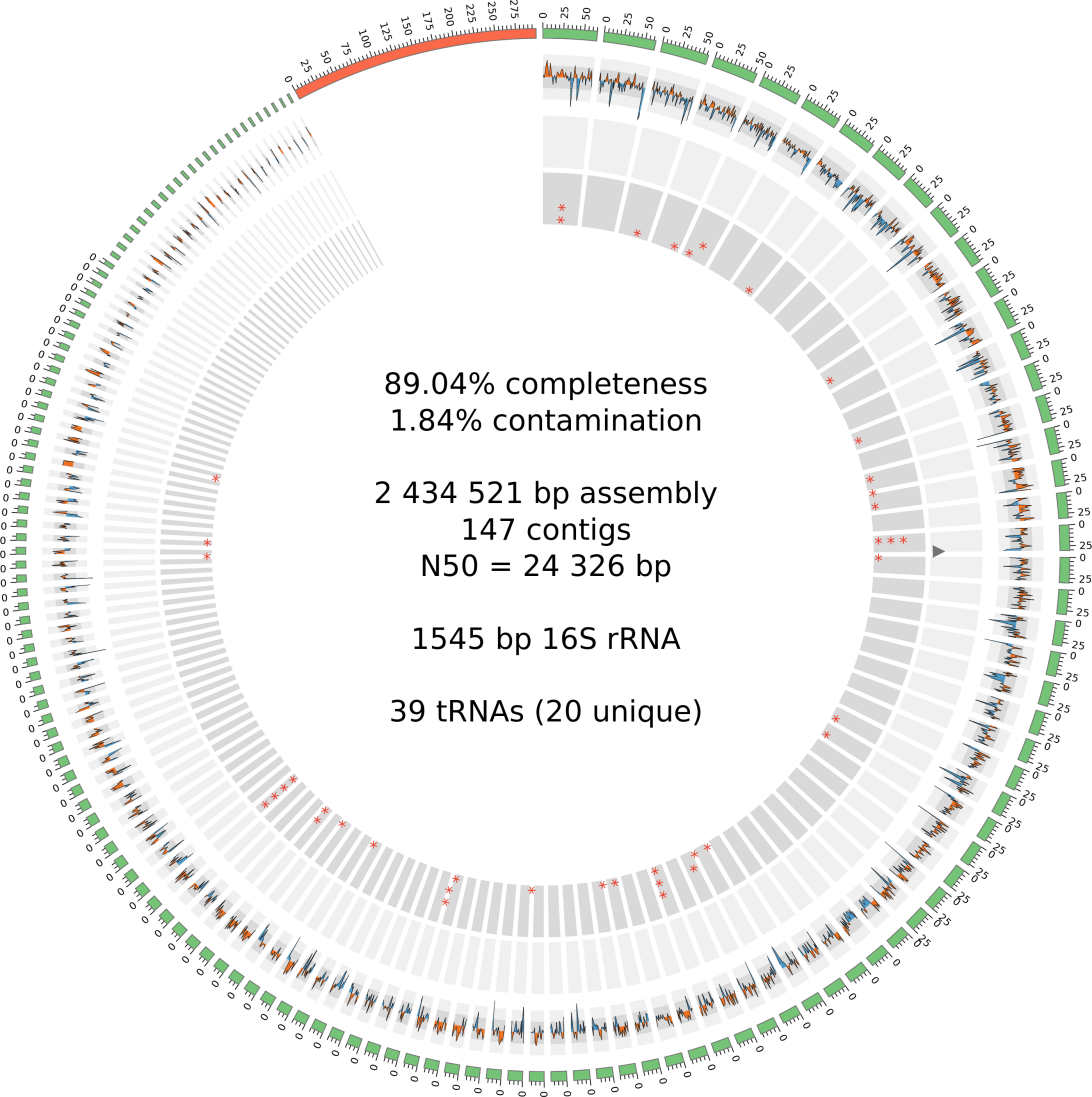
From the outer to inner ring of this Circos metagenome-assembled genome plot: green bars represent the contigs of this MAG, while the red bar is the approximate amount of DNA missing, with ticks indicating contig length in kbp; GC content of 1,000 bp windows from the mean GC of the entire MAG; the location of the 16S rRNA gene (triangle); and the location of tRNA genes (asterisk). This plot was generated with Koonkie Inc. circos_mag software (https://github.com/Koonkie-Cloud-Services/circos_mag) and Circos (9).

One 2,434,521 bp bin showed a 7.10% alignment rate with the metagenome, comprising 147 contigs with an *N*_50_ of 24,326 bp and 2,578 open reading frames (ORFs) as identified by Prodigal, a GC content of 64.985%, and completeness and contamination values of 89.04% and 1.84%, respectively (Fig. 1). A total of 1 16S rRNA and 39 tRNA genes were identified, and GTDb-tk classified this metagenome-assembled genome (MAG) as a member of *Rubrobacteraceae*. Its 16S rRNA gene was unclassified when aligned against the SILVA database (8), and NCBI’s 16S rRNA database showed a closest match to *Rubrobacter radiotolerans* strain P 1 (accession no. NR_029191.2) at 94.12% identity; thus, this MAG is likely a member of a novel genus of *Rubrobacteraceae*.

Based on gene annotations, this MAG is probably a facultatively anaerobic heterotroph, with complete glycolysis, pyruvate oxidation, and glyoxylate cycle pathways. Some chlorophyll production genes are present, plus an incomplete reductive pentose phosphate cycle module, similar to other *Rubrobacteraceae* (10).

This MAG also possesses some methylotrophic features, including the *mer* gene (5,10-methylenetetrahydromethanopterin reductase), used for methoxylated aromatic compound degradation in methanogenesis and methoxydotrophy in archaea (11), although other bacteria are known to use methoxylated aromatics for energy conservation (12). This MAG is also capable of synthesizing Factor 420, potentially to produce secondary metabolites/antibiotics (13).

We also identified various stress response genes in this MAG, including ORFs for oxidative stress, UV stress, osmotic stress, desiccation, and sporulation.

## Data Availability

The data for this work are available at NCBI BioProject PRJNA1189542. The whole shotgun metagenome reads are deposited under accession no. SRR31455428, assembled reads under accession no. JBLRVU000000000.1, and the MAG is accessible under accession no. SRR31488317.
